# Serum 25-hydroxyvitamin D_3_, parathyroid hormone and blood pressure in an elderly cohort from Germany: a cross-sectional study

**DOI:** 10.1186/1743-7075-9-20

**Published:** 2012-03-21

**Authors:** Alexandra Jungert, Heinz J Roth, Monika Neuhäuser-Berthold

**Affiliations:** 1 Institute of Nutritional Science, Justus-Liebig-University, Goethestrasse 55, 35390 Giessen, Germany; 2 Endocrinology and Oncology Department, Limbach Laboratory, 69126, Heidelberg, Germany

**Keywords:** 25-Hydroxyvitamin D_3_, Parathyroid hormone, Blood pressure, Elderly

## Abstract

**Background:**

Although several studies indicate a link between vitamin D status and blood pressure (BP), the results are inconsistent. The purpose of this study is to investigate whether in predominantly non-obese elderly people without vitamin D deficiency or very high intact parathyroid hormone (iPTH) levels serum 25-hydroxyvitamin D_3 _[25(OH)D_3_] and iPTH are independently associated with BP.

**Methods:**

Cross-sectional data of 132 non-institutionalised subjects (90 women and 42 men, aged 66- 96 years) from Giessen, Germany, were analysed. Serum 25(OH)D_3 _and iPTH were measured by an electrochemiluminescence immunoassay and BP was determined with a sphygmomanometer. We performed univariate and multiple regression analyses to examine the influence of 25(OH)D_3 _and iPTH on BP with adjustments for age, body composition and lifestyle factors.

**Results:**

While iPTH had no impact on BP, 25(OH)D_3 _was negatively associated with systolic BP in men, but not in women. After splitting the cohort into antihypertensive medication users and non-users, 25(OH)D_3 _was a significant predictor for systolic and diastolic BP only in men not receiving antihypertensive medicine, even after multiple adjustment. Adjustment for 25(OH)D_3 _resulted in an inverse association of iPTH with diastolic BP also only in men without intake of antihypertensive medicine.

**Conclusions:**

In elderly men without vitamin D deficiency and not taking antihypertensive medicine, 25(OH)D_3 _may be a negative determinant of BP, independent of iPTH, body composition and lifestyle factors. Furthermore, iPTH may be an independent negative determinant of diastolic BP in men not taking antihypertensive medicine.

## Background

Vitamin D may play an important role in blood pressure (BP) regulation. Numerous cells within the human body are able to express the vitamin D receptor, including cardiomyocytes, vascular smooth muscle cells, endothelial cells and renin-producing juxtaglomerular cells [1-3].

At present, results regarding the impact of vitamin D status, determined by circulating 25-hydroxyvitamin D concentrations [25(OH)D], on BP or hypertension are inconsistent. While some studies reported a negative association between vitamin D and BP [4-11], other studies failed to confirm a relationship [12-18] or even reported a positive association [19]. Parathyroid hormone (PTH), which rises in case of a low vitamin D status, may also affect BP [17,18,20-22], but studies are also inconsistent. Subjects in previous studies were partially hypertensive patients [16], morbidly obese [12] or suffered from vitamin D deficiency [23] or primary hyperparathyroidism [22]. Some of the previous studies reported non-fasting measurements [13,17,20], focused on either women or men [5,18] or did not control for potential confounders, such as age, estimated glomerular filtration rate (eGFR), body composition, nutrient intake, use of vitamin D supplements, antihypertensive medication, sun exposure, physical activity or smoking [4,13,22], which may be associated with vitamin D status, PTH and BP. In addition, previous studies often concentrated exclusively on vitamin D or PTH without considering the interaction between both [6-10].

Elderly people are at risk of developing hypertension [24] and of suffering from vitamin D deficiency because of age-related declines in endogenous vitamin D synthesis, sun exposure and dietary intake [25]. Therefore, and in view of the inconsistent results of previous studies, the primary objective of our study was to test whether 25-hydroxyvitamin D_3 _[25(OH)D_3_] or intact PTH (iPTH) contribute to BP in non-institutionalised elderly people, independently of each other and potential confounding factors, such as age, eGFR, body composition, sun exposure, physical activity, vitamin D and calcium intake, alcohol consumption and smoking behaviour. Furthermore, we analysed whether differences regarding the impact of 25(OH)D_3 _and iPTH on BP exist between subjects receiving antihypertensive drugs and subjects without such a medication.

## Methods

### Subjects

Subjects were participants of the GISELA study, a prospective cohort study in which the nutrition and health status of senior citizens from Giessen, Germany (50°35' North) have been observed at annual intervals since 1994 and biannual intervals since 1998. For enrolment, subjects had to be at least 60 years of age and physically mobile. All investigations took place in the Institute of Nutritional Science in Giessen from July to October. After subjects had become familiar with the trial procedure, written informed consent was obtained from each participant. The study protocol was approved by the Ethical Committee of the Faculty of Medicine at the Justus-Liebig-University, Giessen.

The present investigation reports cross-sectional data from the GISELA study obtained in 2008. Subjects with incomplete data were excluded (*n *= 118), as were individuals who suffered from chronic kidney disease (*n *= 4) or oedema (*n *= 6) or who took diuretics (*n *= 8). Seven subjects were identified as outliers regarding their 25(OH)D_3 _and iPTH measurements and/or the residuals of the regression analyses and were therefore not included. After these exclusions, of the 275 elderly people who took part in the follow-up in 2008, data from 90 women and 42 men remained for the following analysis.

### Anthropometric data and body composition

Body weight, body height, body mass index (BMI), waist-to-hip ratio (WHR), waist circumference (WC) and hip circumference were determined as described elsewhere [26]. Body composition was recorded by a single-frequency (50 kHz) bioelectrical impedance analyser (Akern-RJL BIA 101/S, Data Input, Frankfurt, Germany) according to the instructions of the manufacturer and the predictive formula from Roubenoff et al. [27]. This equation was chosen because it was derived from a reference population similar in age to our study subjects and with the same measurement conditions. Moreover, this equation has been validated against dual-energy X-ray absorptiometry (DXA) body composition measurements.

### Lifestyle factors

Nutritional intake was determined using a three-day estimated dietary record, which was developed and validated for the GISELA study [28]. Smoking behaviour, time spent outdoors as indicator for sun exposure, physical activity patterns and further data, such as age, diseases, medications and supplement intake, were collected using self-administered questionnaires. Smoking behaviour was coded as a dummy variable in two categories: never-smokers were allocated to category one, while category two comprised both current smokers and ex-smokers. The use of antihypertensive drugs and the intake of vitamin D supplements were also coded as dummy variables (no/yes). The physical activity level (PAL) of each participant was assessed as described elsewhere [26].

### Blood pressure

Arterial BP was measured between 7:00 a.m. and 11:00 a.m. after a rest of at least 5 min via a sphygmomanometer with a cuff and a stethoscope to detect the Korotkoff sound. Each subject was asked to take a seated position, and their arm was supported at heart level. The first Korotkoff sound was defined as systolic BP (SBP), while the last sound was determined as diastolic BP (DBP). Hypertension was defined as SBP > 140 mmHg and/or DBP > 90 mmHg and/or intake of antihypertensive drugs [29].

### Laboratory measurements

Blood samples were collected between 7:00 a.m. and 11:00 a.m. after an overnight fast. After immediate centrifugation, serum aliquots were stored at -70°C for further analysis. Both 25(OH)D_3 _and iPTH were measured by an electrochemiluminescence immunoassay (ECLIA, Modular E170, Roche Diagnostics^®^, Mannheim, Germany) in the Limbach Laboratory, Heidelberg, Germany. The coefficient of variation for the total analytic precision of this assay was ≤ 9.9 % for 25(OH)D_3 _and ≤ 5.9 % for iPTH. The lower detection limits of this assay were 10.0 nmol/L for 25(OH)D_3 _and 0.127 pmol/L for iPTH. More details are available from Roche Diagnostics [30,31].

We defined 25(OH)D_3 _levels < 25.0 nmol/L as vitamin D-deficient. Due to the lack of an international consensus on an optimal 25(OH)D_3 _status, we applied two cut-off values as adequate (≥ 50 nmol/L and ≥ 75 nmol/L). Serum creatinine was measured by photometric detection (Shimadzu UV-160A) according to the Jaffé reaction [32]. The eGFR, which served as a marker of the kidney function, was calculated with the simplified Modification of Diet in Renal Disease study (MDRD) formula: eGFR (mL/min/1.73 m^2^) = 186 × (serum creatinine mg/dL)^-1.154 ^× age^-0.203 ^× (0.742 if female) [33].

### Statistical analysis

The characteristics of the study participants were expressed as median and 5^th ^to 95^th ^percentile due to non-normally distributed data. Depending on the sample size, normal distribution was tested by the Shapiro-Wilk test and by the Kolmogorov-Smirnov test with the Lilliefors correction and by visual inspection of histograms. Descriptive characteristics were compared between sexes via the Mann-Whitney *U *test for continuous variables. The χ^2 ^test or, alternatively, Fisher's exact test was used for categorical variables. The cohort was split by BP into two groups such that normotensive subjects were compared with hypertensive participants regarding the prevalence of vitamin D insufficiency by means of the Fisher's exact test. In addition, subjects under antihypertensive medication were compared to subjects free of antihypertensive treatment regarding BP values, anthropometric data, % TBF, 25(OH)D_3_, iPTH as well as the prevalence of vitamin D insufficiency using the Mann-Whitney *U *test and the Fisher's exact test when appropriate.

We examined associations between 25(OH)D_3 _and iPTH as well as associations of 25(OH)D_3_, iPTH, eGFR, age, parameters of anthropometry and body composition, and lifestyle factors with BP by univariate regression analyses. Those variables that exhibited a significant association with either SBP or DBP in univariate analyses were considered as confounding variables in the sex-specific multiple regression analyses with SBP and DBP as dependent variables and 25(OH)D_3 _and iPTH as independent variables, respectively. In addition, we conducted a subgroup analysis by comparing participants taking antihypertensive medicine with subjects without such a treatment. Because there were no major changes in the results when 25(OH)D_3 _and iPTH were logarithmically transformed, we report only the non-transformed data. All statistical analyses were conducted with SPSS 18.0 for Windows (SPSS Inc., Chicago, USA). The significance level was set at *P *< 0.05. All tests were two-tailed.

## Results

### Characteristics of the study subjects

The characteristics of the study population are summarised in Table 1. No significant differences between sexes were observed in BP, 25(OH)D_3_, iPTH, age, BMI, PAL, sun exposure, vitamin D and calcium intake and in the percentage of subjects who received antihypertensive drugs or who suffered from elevated BP. Men had higher values for WC, WHR, eGFR and alcohol consumption and included a lower percentage of never-smokers than women, whereas women had a higher % TBF. The median vitamin D intake of both women and men did not meet the current recommendation of 20 μg vitamin D per day [34].

**Table 1:** Descriptive characteristics of the study population


	**Women (*n *= 90)**		**Men (*n *= 42)**		
		
	**Median**	**P_5_, P**_**95**_	**Median**	**P_5_, P_95_**	***P ***^**a)**^

Age (y)	75.5	68.0-86.5	76.0	70.0-84.7	0.321
Body mass index (kg/m^2^)	26.9	21.1-34.5	26.3	22.9-32.3	0.456
Waist circumference (cm)	90.0	71.6-108.4	99.0	84.6-113.7	< 0.0001
Waist-to-hip ratio	0.85	0.75-0.93	0.95	0.86-1.06	< 0.0001
Total body fat (%)	42.4	32.6-50.2	28.9	21.6-37.9	< 0.0001
25(OH)D_3 _(nmol/L)	59.4	40.5-90.6	66.9	39.9-88.8	0.132
iPTH (pmol/L)	4.5	2.3-7.4	4.1	1.8-8.3	0.315
Systolic blood pressure (mmHg)	140.0	110.0-167.8	130.0	110.0-181.9	0.327
Diastolic blood pressure (mmHg)	72.0	60.0-90.0	70.0	60.0-92.4	0.172
eGFR (mL/min/1.73 m^2^)	56.2	43.1-71.3	63.7	46.5-80.4	< 0.0001
Vitamin D intake (μg/d)	2.5	0.3-10.1	3.3	1.0-11.3	0.149
Calcium intake (g/d)	1.0	0.5-1.7	1.0	0.6-1.6	0.961
Alcohol intake (g/d)	2.3	0.0-16.1	5.5	0.0-30.0	0.013
Sun exposure (min/d)	120.0	40.0-360.0	150.0	23.8-394.0	0.071
Physical activity level	1.7	1.5-1.9	1.6	1.4-1.9	0.319
Current or ex-smokers, n (%)	21	23.3	30	71.4	< 0.0001
Vitamin D supplement users, n (%)	13	14.4	2	4.8	0.143
Elevated blood pressure, n (%)^ b)^	38	42.2	15	35.7	0.569
Antihypertensive drugs, n (%)	55	61.1	24	57.1	0.706

Abbreviations: P_5_, 5th percentile; P_95_, 95th percentile; 25(OH)D_3_, 25-hydroxyvitamin D_3_; iPTH, intact parathyroid hormone; eGFR, estimated glomerular filtration rate.

^a) ^Mann-Whitney *U *test for continuous variables and χ^2 ^test for categorical variables.

^b) ^Elevated blood pressure was defined as SBP > 140 mmHg and/or DBP > 90 mmHg.

None of the subjects had a serious vitamin D deficiency (25(OH)D_3 _< 25 nmol/L). However, 23.3 % (*n *= 21) of the women and 21.4 % (*n *= 9) of the men had 25(OH)D_3 _levels < 50 nmol/L, while 16.7 % (n = 15) of the female and 21.4 % (*n *= 9) of the male participants had a 25(OH)D_3 _status of ≥ 75 nmol/L. Hyperparathyroidism, which was defined as iPTH levels > 6.9 pmol/L, had 6.7 % (n = 6) of the women and 9.5 % (*n *= 4) of the men. In the univariate regression analysis using 25(OH)D_3 _as the independent and iPTH as the dependent variable, an inverse association was observed only in women (women: *β *= -0.297; *P *= 0.004 and men: *β *= -0.150; *P *= 0.342).

In women, SBP, DBP, anthropometric data (except for WHR) and % TBF were significantly higher in subjects under antihypertensive medication compared to women free of antihypertensive treatment (all *P *< 0.01), whereas men showed no such differences based on antihypertensive treatment (all *P *> 0.05). Neither 25(OH)D_3 _nor iPTH were significantly different between BP medication users and non-users in either sex (all *P *> 0.200).

### Prevalence of vitamin D insufficiency and blood pressure

Hypertension had 70.0 % of the women and 71.4 % of the men. The prevalence of vitamin D insufficiency did not differ between hypertensive and normotensive subjects, independent of using the 50 nmol/L (women: 23.8 % vs. 22.2 %, *P *= 1.000 and men: 26.7 % vs. 8.3 %, *P *= 0.247) or 75 nmol/L (women: 79.4 % vs. 92.6 %, *P *= 0.215 and men: 86.7 % vs. 58.3 %, *P *= 0.090) cut-off value to define an optimal vitamin D status. In addition, no differences between participants who were under antihypertensive medication and participants without antihypertensive treatment were found with respect to the prevalence of vitamin D insufficiency, independent of using the 50 nmol/L (women: 23.6 % vs. 22.9 %, *P *= 1.000 and men: 20.8 % vs. 22.2 %, *P *= 1.000) or 75 nmol/L (women: 78.2 % vs. 91.4 %, *P *= 0.147 and men: 83.3 % vs. 72.2 %, *P *= 0.462) cut-off value.

### Univariate and multiple regression analyses regarding the association between vitamin D status, intact parathyroid hormone and blood pressure

Table 2 presents the results of the univariate regression analyses of SBP and DBP with relevant parameters. In women, SBP was positively associated with BMI, WC, % TBF and antihypertensive medication, whereas DBP was negatively associated with age and positively associated with antihypertensive treatment. In men, SBP was negatively influenced by 25(OH)D_3 _and positively associated with WHR, whereas DBP was inversely linked to calcium intake and positively associated with WC and WHR.

**Table 2:** Univariate linear regression analyses between blood pressure and other parameters ^a)^


	**Women (*n *= 90)**		**Men (*n *= 42)**	

	**SBP**	**DBP**	**SBP**	**DBP**

SBP (mmHg)	-	0.516 ****	-	0.698 ****
25-Hydroxyvitamin D_3 _(nmol/L)	-0.068	-0.005	-0.388 *	-0.235
Intact parathyroid hormone (pmol/L)	-0.007	-0.087	0.101	0.088
eGFR (mL/min/1.73 m^2^)	-0.019	0.123	-0.139	0.060
Age (y)	-0.026	-0.300 **	0.099	0.088
Body mass index (kg/m^2^)	0.276 **	0.154	0.229	0.211
Waist circumference (cm)	0.230 *	0.090	0.282	0.342 *
Waist-to-hip ratio	0.167	0.019	0.411 **	0.423 **
Total body fat (%)	0.382 ***	0.194	0.012	0.071
Vitamin D intake (μg/d)	-0.019	-0.169	0.082	-0.024
Calcium intake (mg/d)	-0.086	-0.027	-0.105	-0.365 *
Alcohol intake (g/d)	-0.059	0.112	-0.039	0.158
Sun exposure (min/d)	-0.032	-0.078	0.021	-0.066
Physical activity level	-0.158	-0.194	-0.068	-0.095
Current or past smoking ^b)^	-0.103	-0.116	0.136	-0.007
Antihypertensive medication^ b)^	0.332**	0.281**	0.230	0.131
Vitamin D supplement use^ b)^	-0.031	0.041	0.010	0.098

Abbreviations: SBP, systolic blood pressure; DBP, diastolic blood pressure; eGFR, estimated glomerular filtration rate.

^a) ^Univariate regression analyses with SBP and DBP as dependent variables. The shown values represent the standardised coefficient *β*. * *P *< 0.05; ** *P *< 0.01; *** *P *< 0.001; ****P < 0.0001.

^b) ^Dummy variable (no/yes).

The results of the multiple regression analyses for each sex are given in Table 3. Because of the observed collinearity of WC, WHR, BMI and % TBF (data not shown), the variable that showed the strongest association with SBP or DBP (Table 2) was added as an independent variable to the respective model. Consequently, % TBF was included as an independent variable in the female model, while WHR was integrated in the model for men. After adjustment of BP for age, % TBF, antihypertensive medication and mutual adjustment for iPTH or 25(OH)D_3_, neither 25(OH)D_3 _nor iPTH predicted BP in women. In men, even after adjusting BP for iPTH, WHR and calcium intake, 25(OH)D_3 _remained an independent negative predictor of SBP. Besides 25(OH)D_3_, WHR was also a predictor of SBP, and together they accounted for approximately 21.9 % of the variance of SBP.

**Table 3:** Multiple regression analyses using inclusion procedure of factors possibly associated with blood pressure ^a)^


**Women (*n *= 90)**	**SBP (mmHg)**			**DBP (mmHg)**		
	
	**B**	***β***	***P***	**B**	***β***	***P***

Intercept	93.689		0.013	116.202		< 0.0001
25(OH)D_3 _(nmol/L)	-0.059	-0.046	0.669	-0.067	-0.104	0.334
iPTH (pmol/L)	-0.204	-0.017	0.871	-0.455	-0.075	0.468
Age (y)	0.010	0.003	0.976	-0.532	-0.327	0.002
BP medication	8.240	0.225	0.042	5.365	0.294	0.009
TBF (%)	1.029	0.292	0.011	0.033	0.019	0.866
Corr. R^2^		0.141			0.143	

**Men (*n *= 42)**	**SBP (mmHg)**			**DBP (mmHg)**		
	
	**B**	***β***	***P***	**B**	***β***	***P***

Intercept	62.674		0.186	43.040		0.044
25(OH)D_3 _(nmol/L)	-0.503	-0.356	0.015	-0.125	-0.198	0.160
iPTH (pmol/L)	-0.725	-0.068	0.653	-0.049	-0.010	0.945
WHR	117.728	0.387	0.013	49.992	0.366	0.017
Calcium (mg/d)	-0.003	-0.050	0.729	-0.010	-0.320	0.028
Corr. R^2^		0.219			0.243	

Abbreviations: SBP, systolic blood pressure; DBP, diastolic blood pressure; 25(OH)D_3_, 25-hydroxyvitamin D_3_; iPTH, intact parathyroid hormone; BP medication, antihypertensive medication as dummy variable (no/yes); TBF, total body fat; WHR, waist-to-hip ratio. ^a) ^Multiple linear regression analyses with SBP or DBP as dependent variables. The results of the linear regression analyses are expressed in terms of B (the unstandardised coefficient), *β *(the standardised coefficient), and the adjusted coefficient of determination (corr. R^2^).

**Figure 1:**
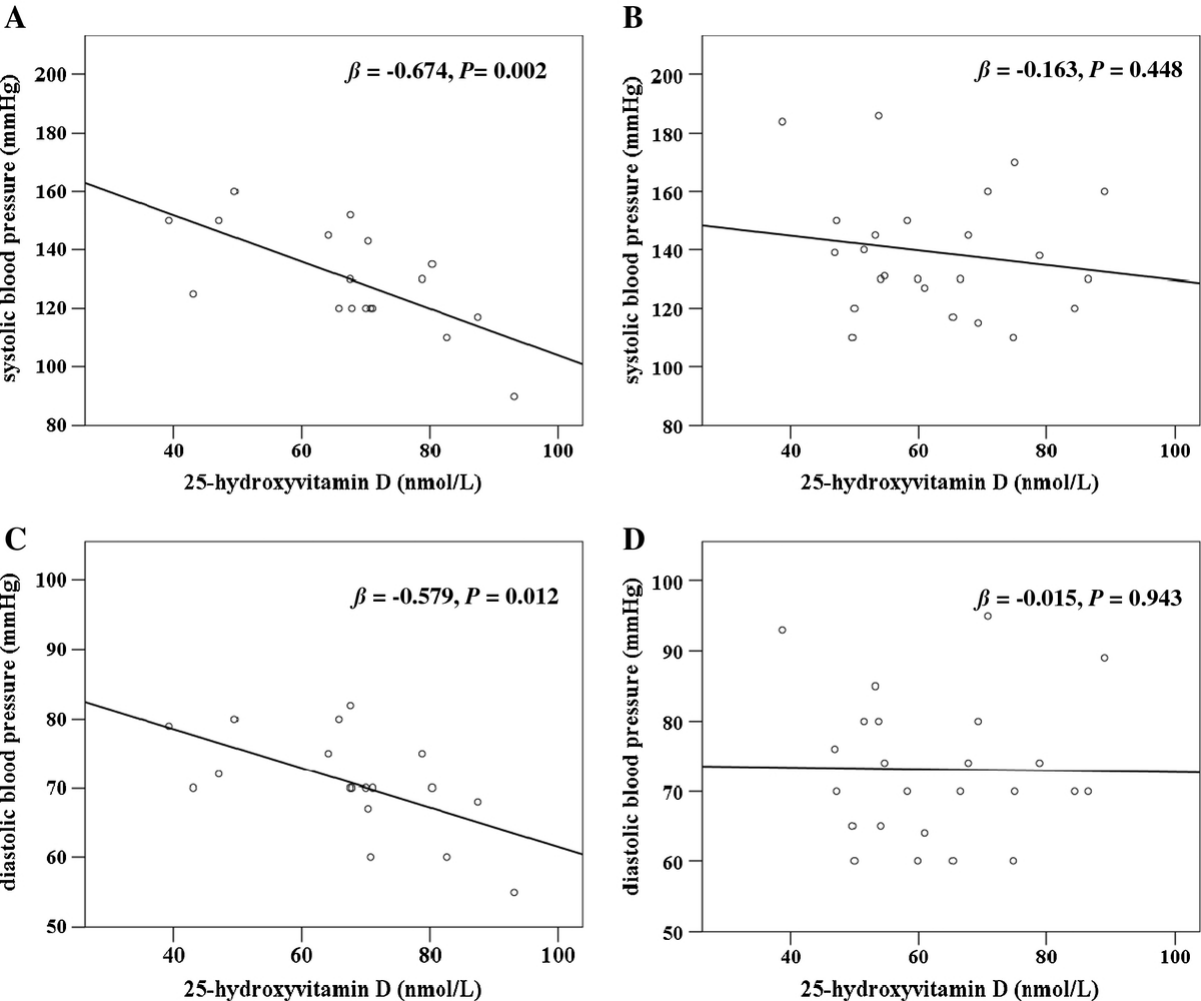
**Associations of 25-hydroxyvitamin D_3 _with blood pressure in men separated by the use of antihypertensive medicine**. A) Association between 25-hydroxyvitamin D_3 _and systolic blood pressure in men without medication. B) Association between 25-hydroxyvitamin D_3 _and systolic blood pressure in men with medication. C) Association between 25-hydroxyvitamin D_3 _and diastolic blood pressure in men without medication. D) Association between 25-hydroxyvitamin D_3 _and diastolic blood pressure in men with medication. The *P* values and the standardised coefficients *β *were calculated by univariate regression analyses.

In men, but not in women, BP medication-users and non-users differed regarding the association between 25(OH)D_3_, iPTH and BP (Table 4): In men, significant inverse associations between 25(OH)D_3 _and both SBP and DBP were only present in the group free of antihypertensive treatment. Serum iPTH had no impact on BP until adjustment for 25(OH)D_3 _was performed, whereupon a negative association between iPTH and DBP was only found in subjects free of antihypertensive treatment. The linear relationships of 25(OH)D_3 _with SBP and DBP in men separated by the intake of antihypertensive medicine are illustrated in Figure 1.

**Table 4:** Associations of 25-hydroxyvitamin D_3 _and parathyroid hormone with blood pressure in antihypertensive medication users and non-users ^a)^


**Women (*n *= 90)**		**BP medication users (*n *= 55)**		**BP medication non-users (*n *= 35)**	
		
		**SBP (mmHg)**	**DBP (mmHg)**	**SBP (mmHg)**	**DBP (mmHg)**

25(OH)D_3 _(nmol/L)	Model 1	-0.119	-0.122	-0.069	0.201
	Model 2	-0.085	-0.146	0.077	0.133
	Model 3	-0.103	-0.164	0.197	0.158

iPTH (pmol/L)	Model 1	-0.062	-0.088	0.202	-0.004
	Model 2	-0.077	-0.066	0.132	-0.022
	Model 3	-0.096	-0.097	0.215	0.045

**Men (*n *= 42)**		**BP medication users (*n *= 24)**		**BP medication non-users (*n *= 18)**	
		
		**SBP (mmHg)**	**DBP (mmHg)**	**SBP (mmHg)**	**DBP (mmHg)**

25(OH)D_3 _(nmol/L)	Model 1	-0.163	-0.015	-0.674 **	-0.579 *
	Model 2	-0.207	-0.088	-0.619 *	-0.529 *
	Model 3	-0.207	-0.088	-0.843 **	-0.785 **

iPTH (pmol/L)	Model 1	0.127	0.185	0.010	-0.194
	Model 2	-0.015	0.054	-0.053	-0.144
	Model 3	-0.016	0.053	-0.449	-0.512 *

Abbreviations: BP medication, antihypertensive medication; SBP, systolic blood pressure; DBP, diastolic blood pressure; 25(OH)D_3_, 25-hydroxyvitamin D_3_; iPTH, intact parathyroid hormone.

^a) ^Multiple linear regression analyses with SBP and DBP as dependent variables and 25(OH)D_3 _and iPTH as independent variables after splitting the cohort into antihypertensive medication users and non-users, respectively. The results are expressed in terms of the standardised coefficient *β*. Model 1: unadjusted association. Model 2: model 1 additionally adjusted for waist-to-hip ratio and calcium intake (mg/d) in men and age (y) and total body fat (%) in women. Model 3: model 2 plus mutual adjustment for iPTH (pmol/L) and 25(OH)D_3 _(nmol/L) in both the male and female model, respectively. * *P *< 0.05; ** *P *< 0.01.

## Discussion

This is the first investigation in a non-institutionalised elderly German cohort without vitamin D deficiency showing an independent association of 25(OH)D_3 _with SBP in elderly men by considering a variety of potential confounding factors including iPTH. Whether this association reflects a causal relationship is open to discussion because of the cross-sectional character of our study. With regard to the mechanism of how the vitamin D endocrine system acts as a regulator of BP several theories are currently being discussed, including the inhibition of the renin-angiotensin system and the enhancement of insulin sensitivity by vitamin D as well as the direct impact of vitamin D on vasculature and heart muscle [1,2,35]. In a population-based study, a low 25(OH)D status has been linked to increased heart rate and rate-pressure product, such that an enhancement of cardiac work due to a suboptimal vitamin D status can be assumed [10]. However, the observed impact of the vitamin D status on BP could also be mediated by PTH [17,20]. Primary hyperparathyroidism has been linked to increased intima-media thickness of the carotid artery as well as carotid stiffness [22]. Furthermore, elevated BP could increase the urinary excretion of calcium, thereby leading to lower serum calcium, which consequently up-regulates PTH secretion [14]. In our investigation, no associations were present between iPTH and BP in the unstratified analysis independent of sex and multiple adjustment, which is in accordance with some [5] but not all previous studies [11,17,18,21]. There is evidence that the association of iPTH with BP is more pronounced in subjects with elevated iPTH and/or subjects with low calcium intake [13]. As the median calcium intake of our cohort met the current recommendations of 1 g per day [36] and subjects with extreme iPTH values were excluded, this may explain why we did not observe an association between iPTH and BP.

We found an inverse association between 25(OH)D_3 _and iPTH in females, but not in males, which may be attributed to hormonal differences, the lower sample size of men and/or the higher % TBF in women. The latter may result in a higher sequestration of 25(OH)D_3 _in the adipose tissue, which consequently up-regulates iPTH secretion. The inverse association between 25(OH)D_3 _and iPTH supports the requirement of a mutual adjustment to evaluate independent effects of vitamin D and iPTH on BP. Some studies have addressed the effect of both 25(OH)D and PTH on BP in an elderly cohort [4,17,18]; however, the majority of these studies performed no mutual adjustment for 25(OH)D and PTH. In two studies, 25(OH)D was not significantly associated with BP, while elevated PTH was significantly related to higher BP [17,18]. The authors argued that the reason for the missing association of 25(OH)D with BP is perhaps attributed to the high vitamin D status of their participants [17,18]. It should be noticed that the median vitamin D status of our subjects was also well in the range considered as sufficient. As in our study, Almirall et al. [4] found an association of 25(OH)D with SBP, but not with DBP, after adjustment for several covariates; however this study included predominantly vitamin D-insufficient [25(OH)D < 50 nmol/L] individuals and did not control for PAL, sun exposure, vitamin D intake and iPTH.

There are some studies in younger study populations, which mutually adjusted for 25(OH)D and PTH. Jorde et al. [37] reported a significant inverse cross-sectional association of 25(OH)D quartiles with SBP in ≥ 25 year-old subjects of the Tromsø Study, whereas the association between 25(OH)D quartiles and DBP failed to reach the significance level after multiple adjustment. In agreement with our results, the additional inclusion of PTH in the multiple regression models by Jorde et al. [37] did not alter the results. In the NHANES 2003-2006, both 25(OH)D and PTH were associated with BP and the prevalence of hypertension in participants aged ≥ 20 years not taking antihypertensive medicine, but the association of 25(OH)D with BP attenuated when adjusted for PTH [11]. He and Scragg [20] reported a non-significant association between 25(OH)D and BP in subjects aged ≥ 20 years after controlling for both PTH and BMI, while the relation of PTH with BP was not considerably affected by 25(OH)D and BMI. Obesity is considered as a risk factor for hypertension [38] and may also be related to the vitamin D endocrine system [39]. Therefore, body composition can be a confounding factor in the associations between vitamin D, PTH and BP [20,23]. In our male participants, body composition as well as iPTH and lifestyle factors had no impact on the association between 25(OH)D_3 _and SBP, which suggests that the underlying mechanism in the association between vitamin D and BP is independent of these confounding variables.

Our results support the hypothesis that the effect of vitamin D on BP is more pronounced for SBP than for DBP [9,37,40]. Interestingly, we found an independent impact of 25(OH)D_3 _on SBP and DBP in the men without antihypertensive treatment, but not in those under antihypertensive medication. Moreover, iPTH was inversely linked to DBP in men without antihypertensive treatment after additional adjustment for 25(OH)D_3_, which has not been reported before. In accordance with our results, Scragg et al. [10] found no association between 25(OH)D and SBP in a subgroup analysis with participants taking BP lowering drugs, whereas in the entire cohort a significant association was observed. In contrast, others [17,18] found no significant association between 25(OH)D and BP even after exclusion of subjects using antihypertensive medication, whereas PTH remained positively associated with BP. In one study, the association between PTH and BP in elderly Chinese men was stronger when subjects using antihypertensive medication were excluded [18]. Almirall et al. [4] reported a correlation of 25(OH)D with BP in subjects with and without antihypertensive treatment; however, this association was slightly stronger in the non-treated group. The reason why the association between 25(OH)D_3 _and BP as well as the relation between iPTH and DBP was only present in the non-treated group of elderly men in our study requires further investigation. Although a false-positive finding cannot be ruled out, one possible explanation could be that antihypertensive medication includes often inhibitors of the renin-angiotensin system, so that vitamin D may have no additional effect on BP in subjects taking antihypertensive drugs. The unexpected negative impact of iPTH on DBP in our study might be due to the enhanced production of 1,25-dihydroxyvitamin D_3_, which possibly functions as an inhibitor of the renin-angiotensin system [1,2]. The question arises why we did not obtain the same findings in the women. The observed sex differences may be attributed to hormonal differences and/or the fact that significant differences in BP and % TBF between subjects with and without antihypertensive medication were only present in women. The higher BP in women under antihypertensive medication may indicate that the antihypertensive treatment so far was less efficient in the female subjects. Because we have no information on the brand, duration or dosage of antihypertensive drugs, possible differences between sexes regarding these drug-related data may also be conceivable explanations. The women might have taken other drugs than men, e.g. beta blocker instead of inhibitors of the renin-angiotensin system. Finally, women and men differed substantially with respect to their predictors of BP. Altogether, these differences might explain why the 25(OH)D_3 _status was only associated with BP in men.

In the present study, neither the estimated nutritional vitamin D intake nor the assessed supplemental vitamin D usage did affect BP, which is in accordance with the literature [13,41,42]. The missing associations of vitamin D intake with 25(OH)D_3 _and iPTH (data not shown) and the low amount of vitamin D intake in our study may be responsible for this observation. Instead, we found that the calcium intake was an independent negative determinant of DBP in men, as noted in other studies [42]. One can assume that in case of an adequate calcium intake and sufficient sun exposure, the habitual vitamin D intake may have no additional effect on BP. In addition, contrary to some other studies [43], daily alcohol intake was not associated with BP in the present investigation, which may be attributed to the relatively low median intake level of our subjects, and/or, possibly, due to the fact that our analysis was not stratified by type of alcoholic beverages, patterns of drinking or acute vs. chronic effects.

Our study has some limitations, including the cross-sectional design, which limits our ability to establish causal relationships. Due to the small sample size, it is possible that some associations were classified as not statistically significant because of a type II error. Participants in this study were volunteers, had a higher educational level and were more aware of health issues than their peers in the general German population [26]. Further limitations are the use of self-reported data, the indirect estimates of sun exposure and physical activity and missing data on type and the exact dosage and duration of vitamin D supplements as well as antihypertensive drugs. We have no information on serum calcium levels, which were linked to BP [40]. However, other studies have shown, that the association between 25(OH)D and BP may be independent of serum calcium [9].

## Conclusion

In conclusion, 25(OH)D_3 _may be a negative determinant of BP in elderly men, especially in those not taking antihypertensive medicine. On the basis of our results, it can be supposed that this effect is independent of iPTH, body composition and lifestyle factors. Furthermore, iPTH may be an independent negative determinant of DBP in subjects not taking antihypertensive medicine. Our findings indicate that an increase in serum 25(OH)D_3 _levels in elderly men, even without vitamin D deficiency, might contribute to the prevention of hypertension.

## Abbreviations

BP: blood pressure; 25(OH)D_3_: 25-hydroxyvitamin D_3_; iPTH: intact parathyroid hormone; eGFR: estimated glomerular filtration rate; SBP: systolic blood pressure; DBP: diastolic blood pressure; GISELA: longitudinal study on nutrition and health status in senior citizens of Giessen: Germany; NHANES: national health and nutrition examination survey; BMI: body mass index; WHR: waist-to-hip ratio; WC: waist circumference; TBF: total body fat; PAL: physical activity level

## Competing interests

The authors declare that they have no competing interests.

## Authors' contributions

AJ developed the study hypothesis, performed the statistical analysis, interpreted the data and wrote the manuscript. HJR analysed the blood samples. MNB designed the study and conducted the research. All authors read and approved the final manuscript.
